# Clinical and Financial Outcomes Due to Methicillin Resistant *Staphylococcus aureus* Surgical Site Infection: A Multi-Center Matched Outcomes Study

**DOI:** 10.1371/journal.pone.0008305

**Published:** 2009-12-15

**Authors:** Deverick J. Anderson, Keith S. Kaye, Luke F. Chen, Kenneth E. Schmader, Yong Choi, Richard Sloane, Daniel J. Sexton

**Affiliations:** 1 Division of Infectious Diseases, Department of Medicine, Duke University Medical Center, Duke Infection Control Outreach Network, Durham, North Carolina, United States of America; 2 Department of Medicine, Detroit Medical Center and Wayne State University, Detroit, Michigan, United States of America; 3 Department of Medicine-Geriatrics, Duke University Medical Center and Geriatric Research Education and Clinical Center (GRECC), Durham VA Medical Center, Durham, North Carolina, United States of America; 4 Center for the Study of Aging and Human Development, Duke University Medical Center, Duke Infection Control Outreach Network, Durham, North Carolina, United States of America; National Institutes of Health, United States of America

## Abstract

**Background:**

The clinical and financial outcomes of SSIs directly attributable to MRSA and methicillin-resistance are largely uncharacterized. Previously published data have provided conflicting conclusions.

**Methodology:**

We conducted a multi-center matched outcomes study of 659 surgical patients. Patients with SSI due to MRSA were compared with two groups: matched uninfected control patients and patients with SSI due to MSSA. Four outcomes were analyzed for the 90-day period following diagnosis of the SSI: mortality, readmission, duration of hospitalization, and hospital charges. Attributable outcomes were determined by logistic and linear regression.

**Principal Findings:**

In total, 150 patients with SSI due to MRSA were compared to 231 uninfected controls and 128 patients with SSI due to MSSA. SSI due to MRSA was independently predictive of readmission within 90 days (OR = 35.0, 95% CI 17.3–70.7), death within 90 days (OR = 7.27, 95% CI 2.83–18.7), and led to 23 days (95% CI 19.7–26.3) of additional hospitalization and $61,681 (95% 23,352–100,011) of additional charges compared with uninfected controls. Methicillin-resistance was not independently associated with increased mortality (OR = 1.72, 95% CI 0.70–4.20) nor likelihood of readmission (OR = 0.43, 95% CI 0.21–0.89) but was associated with 5.5 days (95% CI 1.97–9.11) of additional hospitalization and $24,113 (95% 4,521–43,704) of additional charges.

**Conclusions/Significance:**

The attributable impact of *S. aureus* and methicillin-resistance on outcomes of surgical patients is substantial. Preventing a single case of SSI due to MRSA can save hospitals as much as $60,000.

## Introduction

Surgical site infections (SSIs) are well known to lead to adverse clinical and financial outcomes for patients. The average SSI leads to approximately one week of additional hospitalization and increases the risk of death 2- to 11-fold compared to uninfected surgical patients [1], [2], [3]. In addition, SSIs lead to significant hospital costs. Each SSI costs approximately $12,000–$35,000 (2007 USD), depending on the type of procedure. In total, SSIs cost the US healthcare system approximately $10 billion annually [4].

Methicillin resistant *Staphylococcus aureus* (MRSA) has become the leading cause of SSI in community hospitals [5] and leads to 15% of SSIs reported to the NHSN [6]. To date, only a few studies have specifically examined outcomes related to SSI due to MRSA [7], [8], [9]. These studies have been limited to single institutions, small numbers, and/or a single surgical procedure. The authors of these studies reached conflicting conclusions regarding the impact of methicillin resistance on outcomes among patients with *S. aureus* SSI. Furthermore, only one of these studies [7] evaluated financial outcomes directly attributable to SSI due to MRSA. Thus, financial outcomes due to MRSA SSI are not well described.

An accurate assessment of the financial and human costs of SSI due to MRSA is a necessary first step in justifying and allocating resources for the prevention of MRSA SSI. Indeed, it is important to first develop metrics to assess costs and outcomes before designing studies that assess the benefits and impact of prevention programs. Thus, we conducted a large, multi-center study of multiple surgical procedure types to determine clinical and financial outcomes of SSIs directly attributable to MRSA and methicillin-resistance.

## Methods

### Ethics Statement

All work included in this project was approved by the Institutional Review Boards for all participating hospitals. A waiver of consent was granted given the retrospective nature of the project.

### Study Hospitals

This multi-center matched-outcomes study was conducted at one tertiary care center (750 beds) and six community hospitals (range of hospital sizes = 102 to 305 beds; mean 208 beds). Each of the community hospitals were members of the Duke Infection Control Hospital Network (DICON). The structure and function of DICON have been previously described [10], [11].

SSIs were prospectively identified by trained infection preventionists using CDC definitions and National Healthcare Surveillance Network (NHSN) criteria [12], [13]. Surveillance was identical at all study hospitals and included all operative procedures with only one exception. Surveillance was limited to cardiothoracic, neurosurgical, and orthopedic procedures at DUMC.

### Study Population

The study population for this analysis has been described in detail elsewhere [14]. Briefly, three sets of patients were identified from preexisting prospectively collected databases during the time period from January 1, 1998, through April 1, 2003 (the “study period”): patients with SSI due to MRSA, patients with SSI due to MSSA, and uninfected surgical patients. We compared patients with SSI due to MRSA to two sets of controls: uninfected, matched controls and patients with SSI due to MSSA. Uninfected surgical patients were frequency matched to patients with SSI due to MRSA by type of operative procedure, hospital, and year of procedure. All patients with SSI due to either MRSA or MSSA at the study hospitals during the study period were included. Thus, no matching was performed between patients with SSI due to MRSA and patients with SSI due to MSSA.

### Study Variables

#### Independent variables

Data were abstracted from two sources: prospectively collected surgical surveillance databases and patient records. The following data were prospectively collected and maintained in surgical surveillance databases: patient age, type of procedure, date of surgery, length of procedure, type of procedure, wound classification, American Society of Anesthesiologists (ASA) score, NNIS risk index score, and, if SSI was present, pathogen, anatomic site of infection, and date of culture [15]. Study nurses retrospectively collected gender, race, admission source, insurance, comorbid conditions, preoperative functional status, serum glucose, and antibiotic administration from patient charts.

#### Definitions

Obesity was defined as a body mass index of 30 or more. Peri-operative antimicrobial prophylaxis was considered to be appropriate if an antimicrobial agent recommended by a published guideline was administered 2 hours or less before the surgical incision [16]. The preoperative level of independence as determined by activities of daily living was determined according to the Katz criteria [17]. The overall level of comorbid illness was determined for each study patient by calculating a Charlson score [18]. Acute severity of illness was determined by calculating a McCabe score [19]. Effective antimicrobial therapy was defined as initiation of an antibiotic with in vitro activity against the pathogen within 7 days of diagnosis of SSI.

#### Outcome variables

Outcomes data were obtained from patients chart and the U.S. Social Security Death Index. Four outcome variables were analyzed for the 90-day postoperative period: hospital readmission, mortality (including both in-hospital and outpatient), total hospital days (including readmissions), and hospital charges (including readmissions). All hospital charges were adjusted to reference year 2003 by inflating charges from prior years at a 3% annual rate.

### Statistical Analysis

All statistical analyses were performed using SAS software, version 9.1 (SAS). Continuous variables were compared in bivariable analysis using the Wilcoxon rank sum test or Student t-test. Dichotomous and ordinal variables were compared using the Fisher exact or chi-square tests, where appropriate. The Kaplan-Meier method was used to determine differences in 90-day mortality over time.

Each of the four outcomes was analyzed in two ways: 1) patients with SSI due to MRSA were compared to uninfected controls to determine the impact of SSI due to MRSA on outcomes and 2) patients with SSI due to MRSA were compared to patients with SSI due to MSSA to determine the impact of methicillin-resistance on outcomes. Logistic regression was performed to determine the independent effects of SSI due to MRSA and methicillin-resistance on 90-day readmission rates and 90-day mortality. Linear regression was used to determine the independent effects of SSI due to MRSA and methicillin-resistance on 90-day duration of hospitalization after surgery and 90-day hospital charges (after log transformation of the outcome variable). In addition, linear regression with the least squares means method was used to determine adjusted mean values attributable to SSI due to MRSA and methicillin-resistance for postoperative total hospital days and 90-day hospital charges.

Variables with a p-value≤0.2 in bivariable analysis were included as candidate variables for the multivariable models. Models were derived using backwards selection. Only confounding variables and variables with an adjusted p-value≤0.05 were included in the final models. Confounding variables were identified as variables that, once removed, changed β coefficients by more than 10%.

Variables considered for inclusion in the models were assessed for missing data. Missing data for these variables were imputed using unconditional imputation: imputation of the mean for continuous variables or the mode for categorical variables [20]. If >5% of data for a variable were missing, the variable was tested for bias by creating dummy variables for the imputed data. If the imputation dummy variable was significantly associated with the outcome (e.g., 90-day mortality), then it was left in the final model to control for bias generated by imputation [10].

Finally, because inappropriate therapy for MRSA infections leads to worse outcomes [21], a sensitivity analysis was performed that compared patients with SSI due to MRSA who received appropriate therapy to patients with SSI due to MSSA who received appropriate therapy to see if outcomes were worse among patients with SSI due to MRSA, even if therapy was administered appropriately.

## Results

A total of 278 patients with SSI due to *S. aureus* were identified following 141,345 procedures during the study period (overall rate of SSI due to *S. aureus* = 0.20/100 procedures); 150 patients were diagnosed with SSI due to MRSA (54% of SSI due to *S. aureus*; rate of SSI due to MRSA = 0.11/100 procedures) and 128 patients were diagnosed with SSI due to MSSA (rate of SSI due to MSSA = 0.09/100 procedures). Table 1 summarizes key demographic, clinical, and surgical variables.

**Table 1:** Key characteristics of 150 patients with methicillin-resistant *Staphylococcus aureus* surgical site infections (SSI) compared with 231 uninfected controls and 128 patients with methicillin-susceptible *S. aureus* SSI.^a^

	MRSA SSI N = 150 n(%)	Uninfected Controls N = 231 n(%)	MSSA SSI N = 128 n(%)
***Demographics***			
Age (mean±STD)	62.1±15.4	65.7±15.6	60.4±15.0
Gender (male)	70 (46.7)	121 (52.4)	64 (50.4)
Race (Caucasian)	104 (70.3)	184 (80.4)	98 (77.2)
Admitted from home	103 (76.7)	168 (74.3)	93 (81.6)
Medicaid insurance	16 (11.0)	6 (2.6)^b^	14 (11.5)
***Selected comorbid conditions***			
Charlson≥3	35 (23.3)	33 (14.3)^c^	18 (14.1)^c^
McCabe score on admission of 1	12 (8.2)	4 (1.8)^c^	4 (3.4)
BMI>30	57 (41.3)	59 (26.9)	57 (48.7)
Diabetes mellitus	39 (26.0)	58 (25.1)	29 (22.7)
Congestive heart failure	44 (29.3)	37 (16.0)^b^	23 (18.0)^c^
Cerebrovascular disease	15 (10.0)	18 (7.8)	8 (6.3)
Chronic obstructive pulmonary disease	26 (17.3)	20 (8.7)	27 (21.1)
Renal disease	15 (10.0)	14 (6.1)	6 (4.7)
Use of immuosuppressive medications	17 (12.0)	16 (7.1)	7 (5.9)
***Preoperative functional status***			
No limitations	74 (49.3)	166 (71.9)^b^	84 (65.6)^c^
Need assistance with 3 or more ADLs	46 (30.7)	12 (9.4)^b^	25 (10.8)^b^
***Surgical Characteristics***			
Orthopedic procedure	69 (46.0)	95 (41.1)	56 (43.8)
Cardiothoracic procedure	43 (28.7)	84 (36.4)	44 (34.4)
Procedure performed at tertiary care hospital	94 (62.7)	150 (64.9)	75 (58.6)
Repeat procedure at same operative site	17 (11.6)	24 (10.8)	18 (14.4)
Operative procedure >75^th^ percentile	36 (42.4)	49 (29.7)^c^	16 (25.0)^c^
Wound class >2	16 (10.7)	7 (3.0)^c^	0^a^
ASA score≥3	109 (73.2)	156 (69.6)^c^	94 (75.8)
Serum glucose >200 mg/dL	42 (37.2)	65 (39.6)	30 (33.7)
Antimicrobial prophylaxis administered appropriately	108 (75.5)	176 (77.9)	91 (79.1)
Surgery on same day as hospital admission	74 (49.3)	131 (57.0)^c^	79 (61.7)^c^

A - All percentages were calculated using denominators that excluded missing data. Data were missing for the following variables: McCabe score (4 MRSA SSI, 5 uninfected controls, 10 MSSA SSI), BMI (12 MRSA SSI, 12 uninfected controls, 11 MSSA SSI), use of immunosuppressive medications (8 MRSA SSI, 6 uninfected controls, 10 MSSA SSI), repeat procedure (3 MRSA SSI, 8 uninfected controls, 3 MSSA SSI), operative procedure >75^th^ percentile (65 MRSA SSI, 66 uninfected controls, 64 MSSA SSI), ASA score (1 MRSA SSI, 7 uninfected controls, 4 MSSA SSI), serum glucose (37 MRSA SSI, 67 uninfected controls, 39 MSSA SSI), antimicrobial prophylaxis (7 MRSA SSI, 5 uninfected controls, 3 MSSA SSI), same day procedure (1 uninfected control).

B - p<0.001 compared to patients with SSI due to MRSA.

C - p<0.05 compared to patients with SSI due to MRSA.

Orthopedic and cardiothoracic procedures were the two most common procedure types (Table 1). Approximately 60% of procedures were performed at the tertiary care center (Table 1). Among the community hospitals, an average of 9 SSIs due to MRSA and 9 SSIs due to MSSA were diagnosed. In addition, 23 (15%) of patients with SSI due to MRSA and 17 (13%) patients with SSI due to MSSA were admitted to the ICU prior to the diagnosis of their SSI (p = 0.63). Finally, 107 (71%) patients with SSI due to MRSA and 89 (70%) of patients with SSI due to MSSA received appropriate antimicrobial therapy (p = 0.75).

### Outcomes – Impact of SSI Due to MRSA

All outcomes were more severe among patients with SSI due to MRSA compared with matched uninfected controls in unadjusted analyses (Table 2). Patients with SSI due to MRSA were 30-fold more likely to be readmitted and 7-fold more likely to die within 90 days compared to uninfected controls. Similarly, patients with SSI due to MRSA stayed in the hospital 16 more days and accrued more than $40,000 of additional charges compared to uninfected controls.

**Table 2:** Unadjusted clinical and financial outcomes^a^ of 150 patients with methicillin-resistant *Staphylococcus aureus* surgical site infections (SSI) compared with 231 uninfected controls and 128 patients with methicillin-susceptible *S. aureus* SSI.

	MRSA SSI N = 150 n (%)	Uninfected Controls N = 231 n (%)	Unadjusted Odds Ratio [95% CI]; p-value	MSSA SSI N = 128 n (%)	Unadjusted Odds Ratio [95% CI]; p-value
Died during admission	5 (3.5)	2 (0.9)	4.69 [0.88–25.1]; 0.08	1 (0.8)	4.31 [0.50–37.4]; 0.15
Discharged to^b^:					<0.0001
Home	90 (65.7)	175 (78.5)	0.33 [0.17–0.63]; 0.0005	92 (78.0)	0.54 [0.31–0.95]; 0.03
Facility	47 (34.3)	48 (21.5)	3.06 [1.59–5.84]; 0.0005	26 (22.0)	2.05 [1.16–3.62]; 0.01
***Outcomes within 90-days of procedure***					
Readmitted within 90 days within of procedure b	110 (77.5)	23 (10.2)	30.2 [16.8–54.1]; <0.0001	108 (87.1)	0.51 [0.26–0.98]; 0.04
Dead within 90 days of procedure	25 (16.7)	7 (3.0)	7.20 [2.86–18.1]; <0.0001	9 (7.0)	2.64 [1.19–5.90]; 0.01
Total post-procedure length of hospitalization (days) – median (IQR)	21 (10–32)	5 (3–7)	<0.0001	15 (7–22)	0.003
Hospital charges – median (IQR)^c^	79,029 (38,113–127,846)	38,735 (17,753–60,627)	<0.0001	55,667 (22,201–86,757)	0.001

a P values calculated using Student t test or Wilcoxon rank sum test for continuous variables. P-values, odds ratios, and 95% confidence intervals for categorical variables were calculated using the Cochran-Mantel-Haenszel test (MRSA SSI v. matched-uninfected controls) and the Fisher exact test or chi-square (MRSA SSI v MSSA SSI). All percentages were calculated using denominators that excluded missing data.

b Denominator includes patients who survived their index admissions.

c Financial data were available for 144 cases (96%), 202 (87%) uninfected controls, and 127 (99%) MSSA SSI controls.

Table 3 summarizes independent predictors for each outcome of interest for patients with SSI due to MRSA and matched uninfected controls. Similar to unadjusted analyses, all outcomes were worse among patients with SSI due to MRSA. SSI due to MRSA was independently predictive of readmission within 90 days (OR = 35.0, 95% CI 17.3–70.7), death within 90 days (OR = 7.27, 95% CI 2.83–18.7), longer hospitalization (OR = 4.36, 95% CI 3.31–5.75), and higher hospital charges (OR = 4.44, 95% CI 2.68–7.34) compared to uninfected controls. Of note, need for assistance with ≥3 ADLs was also independently predictive of readmission within 90 days, 90-day mortality, and increased length of hospitalization, but not increased hospital charges.

**Table 3:** Independent Predictors of Post-Operative Adverse Outcomes: Analysis of 150 patients with methicillin-resistant *Staphylococcus aureus* (MRSA) surgical site infections (SSI) compared with 231 uninfected controls to determine the independent effect of SSI due to MRSA on outcomes of surgical patients.

Independent Predictor	Odds Ratio [95% Confidence Interval]
*Readmission within 90 days of surgical procedure* a	
SSI due to MRSA	35.0 [17.3–70.7]
Need assistance with ≥3 ADLs	4.28 [1.52–12.0]
*Death within 90 days of surgical procedure* b	
SSI due to MRSA	7.27 [2.83–18.7]
Need assistance with ≥3 ADLs	6.73 [2.80–16.2]
Age≥65	4.45 [1.41–14.0]
Orthopedic procedure	0.27 [0.10–0.71]
*Increased length of hospitalization during 90 days following surgical procedure* c	
SSI due to MRSA	4.36 [3.31–5.75]
Procedure at tertiary care hospital	1.41 [1.30–1.54]
Need assistance with ≥3 ADLs	1.35 [1.25–1.46]
Post-operative serum glucose >200 mg/dL	1.18 [1.15–1.22]
Orthopedic procedure	0.68 [0.62–0.75]
*Hospital charges during 90 days following surgical procedure* d	
SSI due to MRSA	4.44 [2.68–7.34]
Procedure at tertiary care hospital	2.97 [2.23–3.95]
Coronary artery bypass graft procedure	1.34 [1.26–1.43]
Surgical duration >75^th^ NNIS percentile	1.27 [1.22–1.32]
Procedure on same day as admission	0.75 [0.72–0.79]

a Patients who died during the index admission (n = 23) were excluded from this analysis. Final model controlled for confounding effect of ASA score and contained term for interaction between SSI due to MRSA and Need assistance with ≥3 ADLs. Reference model also included the following variables: sex, history of congestive heart failure, history of cerebrovascular accident, McCabe score = 1, and surgery on same day as admission.

b Reference model also included the following variables: admitted from home, Charlson score ≥3, McCabe score = 1, wound class >2, ASA score ≥3, surgery on same day as admission, serum glucose >200 mg/dL, and repeat procedure at same operative site.

c Final model controlled for confounding effects of Caucasian race, McCabe score = 1, male sex, coronary artery bypass graft procedures, and surgical duration >75^th^ NNIS percentile and contained a term for the interaction of MRSA SSI and need assistance with 3 or more ADLs. Reference model also contained the following variables: BMI≥30, age≥65 years, admission from home, Charlson score ≥3, ASA score ≥3, and repeat procedure at same operative site.

d Final model controlled for confounding effects of ASA score ≥3 and contained an interaction term for the interaction between MRSA SSI and need assistance with 3 or more ADLs and an interaction term for the interaction between MRSA SSI and procedure at a tertiary care hospital. Reference model also contained the following variables: need assistance with 3 or more ADLs, receipt of immunosuppressive medications, McCabe score = 1, post-operative serum glucose >200 mg/dL, and orthopedic procedure.

The mean length of stay independently and directly attributable to SSI due to MRSA was 23 days (95% CI 19.7–26.3) compared to uninfected controls (Table 4). The mean hospital charge independently and directly attributable to SSI due to MRSA was $61,681 (95% 23,352–100,011). In total, the charge attributable to SSI due to MRSA was approximately $19 million for the 7 hospitals.

**Table 4:** Length of stay and hospital charges a within 90 days of surgery attributable to surgical site infection (SSI) due to methicillin resistant *Staphylococcus aureus* (MRSA): SSI due to MRSA compared to uninfected controls.

	Length of Stay Least Squares Mean (IQR)		Charges Least Squares Mean (IQR)	
	Unadjusted	Adjusted b	Unadjusted	Adjusted c
Cases	23.6 (21.7–25.5)	28.3 (25.7–30.8)	105,214 (91,458–118,971)	112,144 (85,850–138,438)
Controls	5.2 (3.7–6.7)	5.2 (3.5–7.0)	47,099 (35,485–58,714)	50,463 (34,551–66,375)
Attributable difference	18.4 (16.0–20.8)	23.0 (19.7–26.3)	58,115 (40,111–76,119)	61,681 (23,352–100,011)

a Charges were normalized to year 2003 by adjusting for inflation at a rate of 0.03% per year.

b Adjusted for procedure at tertiary care hospital, need assistance with ≥3 ADLs, post-operative serum glucose >200 mg/dL, orthopedic procedure, caucasian race, McCabe score = 1, male sex, coronary artery bypass graft procedures, surgical duration >75^th^ NNIS percentile, and contained a term for the interaction of MRSA SSI and need assistance with 3 or more ADLs.

c Adjusted for procedure at tertiary care hospital, coronary artery bypass graft procedure, surgical duration >75^th^ NNIS percentile, procedure on same day as admission, ASA score ≥3, the interaction between MRSA SSI and need assistance with 3 or more ADLs and the interaction between MRSA SSI and procedure at a tertiary care hospital.

### Outcomes – Impact of Methicillin-Resistance

**Figure 1:**
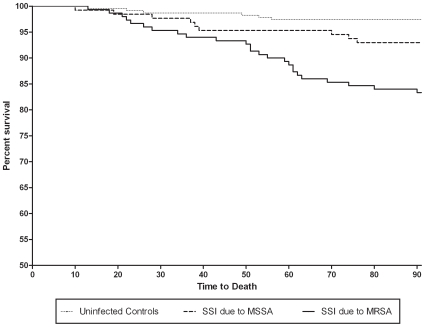
Survival analysis of cases and controls. Kaplan-Meier Survival Analysis of mortality among patients with surgical site infection (SSI) due to methicillin resistant *Staphylcoccus aureus*, SSI due to methicillin-susceptible *S. aureus*, and uninfected controls.

In unadjusted analyses, most outcomes were worse among patients with SSI due to MRSA compared with patients with SSI due to MSSA (Table 2). Patients with SSI due to MRSA were 2.6-fold more likely to die within 90 days following surgery than patients with SSI due to MSSA. Similarly, patients with SSI due to MRSA stayed in the hospital 6 more days and accrued more than $23,000 of additional charges compared patients with SSI due to MSSA. The one exception to this trend was readmission within 90 days of procedure. Patients with SSI due to MRSA were one-half as likely to require readmission within 90 days of procedure as patients with SSI due to MSSA. Ninety-day survival curves for each of the three groups are presented in Figure 1.

Table 5 summarizes independent predictors for each outcome of interest for patients with SSI due to MRSA compared to patients with SSI due to MSSA. Methicillin-resistance was independently predictive of increased length of hospitalization (OR = 1.27, 95% CI 1.22–1.33) and increased hospital charges (OR = 1.20, 95% CI 1.16–1.25) but was not independently associated with increased mortality (OR = 1.72, 95% CI 0.70–4.20). Interestingly, patients with SSI due to MRSA were less likely to be readmitted within 90 days than patients with SSI due to MSSA (OR = 0.43, 95% CI 0.21–0.89). Need for assistance with ≥3 ADLs was associated with increased risk of 90-day mortality while patients who received appropriate administration of peri-operative antimicrobial prophylaxis had lower risk of death than patients who did not.

**Table 5:** Independent Predictors of Post-Operative Adverse Outcomes: Analysis of 150 patients with methicillin-resistant *Staphylococcus aureus* (MRSA) surgical site infections (SSI) compared with 128 patients with methicillin-susceptible *S. aureus* (MSSA) SSI to determine independent effect of methicillin-resistance on patients with *S. aureus* SSI.

Independent Predictor	Odds Ratio [95% Confidence Interval]
*Readmission within 90 days of surgical procedure* a	
Methicillin-resistance	0.43 [0.21–0.89]
Underwent coronary artery bypass grafting	4.35 [1.31–14.5]
Procedure performed at tertiary care facility	2.19 [1.03–4.63]
Admission to ICU prior to infection	0.20 [0.05–0.72]
*Death within 90 days of surgical procedure* b	
Methicillin-resistance	1.72 [0.70–4.20]
Need assistance with ≥3 ADLs	3.79 [1.33–10.8]
Antimicrobial prophylaxis administered appropriately	0.35 [0.14–0.88]
*Increased length of hospitalization during 90 days following surgical procedure* c	
Methicillin-resistance	1.27 [1.22–1.33]
ASA score ≥3	1.65 [1.48–1.84]
Procedure at tertiary care hospital	1.44 [1.34–1.54]
Charlson score ≥3	1.31 [1.23–1.40]
Surgery on same day as admission	0.77 [0.73–0.81]
*Hospital charges during 90 days following surgical procedure* d	
Methicillin-resistance	1.20 [1.16–1.25]
Procedure at tertiary care hospital	1.99 [1.73–2.30]
ASA score ≥3	1.58 [1.42–1.74]
In ICU prior to infection	1.37 [1.26–1.48]
Surgical duration >75^th^ NNIS percentile	1.28 [1.21–1.36]
Surgery on same day as admission	0.65 [0.59–0.71]

a Patients who died during the index admission (n = 23) were excluded from this analysis. Final model controlled for the confounding effect of Medicaid insurance. Reference model also included the following variables: Charlson score ≥3, wound class >2, ASA score ≥3, surgery on same day as admission, post-operative glucose >200 mg/dl, and receipt of effective antimicrobial therapy after infection.

b Final model controlled for the confounding effects of age, ASA score, coronary artery bypass graft procedure, and admission to the ICU prior to infection. Reference model also included the following variables: Medicaid insurance, Charlson score ≥3, post-operative glucose >200 mg/dl, and receipt of effective antimicrobial therapy after infection.

c Final model controlled for the confounding effects of surgical duration >75^th^ NNIS percentile. Reference model also contained the following variables: BMI ≥30, age ≥65 years, admission from home, McCabe score = 1, post-operative glucose >200 mg/dl, coronary artery bypass grafting, orthopedic procedure, and appropriate administration of peri-operative antimicrobial prophylaxis.

d Final model controlled for confounding effects of Charlson score ≥3 and coronary artery bypass graft surgery. Reference model also contained the following variables: age ≥65 years, admission from home, lack of independence with ambulation, McCabe score = 1, post-operative serum glucose >200 mg/dL, and orthopedic procedure.

The mean length of stay independently and directly attributable to methicillin-resistance was 5.5 days (95% CI 1.97–9.11) (Table 6). The mean hospital charge independently and directly attributable to methicillin-resistance was $24,113 (95% 4,521–43,704).

**Table 6:** Length of stay and hospital charges a within 90 days of surgery attributable to surgical site infection (SSI) due to methicillin resistant *Staphylococcus aureus* (MRSA): SSI due to MRSA compared to SSI due to MSSA.

	Length of Stay Least Squares Mean (IQR)		Charges Least Squares Mean (IQR)	
	Unadjusted	Adjusted b	Unadjusted	Adjusted c
SSI due to MRSA	24.3 (21.7–26.8)	23.7 (21.3–26.0)	105,214 (89,558–120,871)	99,466 (86,352–112,580)
SSI due to MSSA	17.4 (14.6–20.2)	18.1 (15.5–20.7)	68,835 (52,164–85,506)	75,353 (61,351–89,355)
Attributable difference	6.86 (3.07–10.4)	5.5 (1.97–9.11)	36,379 (13,509–59,250)	24,113 (4,521–43,704)

a Charges were normalized to year 2003 by adjusting for inflation at a rate of 0.03% per year.

b Adjusted for surgical duration >75^th^ NNIS percentile, ASA score ≥3, procedure at tertiary care hospital, Charlson score ≥3, and surgery on same day as admission.

c Adjusted for surgical duration >75^th^ NNIS percentile, ASA score ≥3, procedure at tertiary care hospital, Charlson score ≥3, surgery on same day as admission, and coronary artery bypass graft surgery.

Sensitivity analyses were performed to determine the impact of appropriate antimicrobial treatment on differences in outcomes among patients with SSI due to MRSA and patients with SSI due to MSSA. Each outcome model was rerun using only the subset of patients who received appropriate therapy. Overall, no differences were noted compared to the results from the full models (data not shown). Specifically, no difference was detected in 90-day mortality for patients with SSI due to MRSA who received appropriate antimicrobial therapy compared with patients with SSI due to MSSA who received appropriate antimicrobial therapy.

## Discussion

Our study represents the largest study to date of outcomes due to SSI due to MRSA. Our findings confirm that SSIs due to MRSA lead to significant patient suffering and provide quantitative estimates of the staggering costs of these infections. SSI due to MRSA led to a 7-fold increased risk of death, a 35-fold increased risk of hospital readmission, more than 3 weeks of additional hospitalization, and more than $60,000 of additional charges compared to uninfected controls.

Numerous studies have evaluated the impact of methicillin-resistance in patients with bloodstream infection (BSIs), yet many of these studies have come to conflicting results [22], [23], [24], [25], [26], [27]. An array of confounding factors have been cited as potential causes for these conflicting conclusions, including patient mix and co-morbid conditions, treatment, severity of illness, and even methods for analysis [28], [29]. The authors of two meta-analyses analyzed data from many of the studies cited above; both concluded that, on the whole, available data suggested that methicillin-resistance is associated with higher mortality among patients with *S. aureus* BSI [7], [30].

The issue is less clear regarding the impact of methicillin-resistance among patients with *S. aureus* SSI. To our knowledge, only three other studies directly compared patients with SSI due to MRSA to patients with SSI due to MSSA in an attempt to determine the attributable impact of methicillin-resistance on outcomes among patients with *S. aureus* SSI [7], [8], [9].

The first study compared 15 patients with mediastinitis due to MRSA to 26 patients with mediastinitis due to MSSA at a single center in France [9]. Patient follow-up was continued for four years. Using multivariable analytic statistical techniques, the authors of this small study concluded that mediastinitis due to MRSA led to a 4.6-fold increase in risk of mortality compared to mediastinitis due to MSSA. No other outcomes were analyzed.

The second study compared 73 patients with mediastinitis due to MRSA to 145 patients with mediastinitis due to MSSA in a single center in France [8]. Outcomes of patients admitted to the ICU with *S. aureus* mediastinitis were analyzed. Methicillin resistance was not an independent predictor of ICU mortality using multivariable analyses. However, mediastinitis due to MRSA was a predictor of a longer duration of mechanical ventilation and ICU stay compared to mediastinitis due to MSSA in an unadjusted statistical analysis.

The third study compared 127 patients with SSI due to MRSA to 173 patients with SSI due to MSSA in two centers (one tertiary care and one community hospital) in North Carolina, USA [7]. Several different types of surgical procedures were included in the analysis, though the majority of procedures were cardiothoracic. In multivariable analyses, methicillin resistance was associated with a 3-fold increase in 90-day mortality, 3 additional days of hospitalization, and $14,000 of additional charges per SSI.

Our multi-center study demonstrated that methicillin-resistance led to longer hospitalization and higher charges among patients with *S. aureus* SSI. Of note, patients with SSI due to MRSA had higher baseline proportions of co-morbid illness than both uninfected controls and patients with SSI due to MSSA. Our outcomes analyses controlled for these differences. Although methicillin resistance led to higher risk of mortality among patients with *S. aureus* SSI in unadjusted analyses, SSI due to MRSA was no longer an independent predictor for risk of mortality compared to SSI due to MSSA after controlling for variables for co-morbid illness, severity of infection, and appropriateness of treatment. These results did not change in our sensitivity analysis limited to patients who received appropriate therapy. However, our Kaplan-Meier analysis suggests that differences may have existed if other time points had been selected, as the mortality curves for patients with SSI due to MRSA and patients with SSI due to MSSA quickly diverged. Nevertheless, the impact of methicillin resistance on outcome of patients who survived was substantial. Our adjusted analyses also demonstrated that methicillin-resistance among patients with *S. aureus* SSI led to approximately 6 additional days of hospitalization and more than $24,000 of additional charges.

Our estimates of the financial burden of SSI due to MRSA are unique. On the whole, SSI due to MRSA led to charges in excess of $19 million for the group of study hospitals. We believe our estimate for the attributable impact of a single SSI due to MRSA of more than $61,000 can be used by administrators and infection control personnel to design and evaluate specific preventative interventions. For example, if an intervention (e.g., decolonization, screening, hiring of one FTE) costs less than $61,000 and leads to the prevention of only one SSI due to MRSA, then this intervention will likely be cost effective for the institution.

Our study has limitations. First, our study included only deep incisional and organ/space infections. Thus, our findings cannot be generalized to superficial incisional SSIs due to MRSA. Deep incisional and organ/space SSIs, however, are more severe and clinically important than superficial SSI. In fact, cost estimates would have been even higher had we included superficial incisional infections in our analysis. Second, our charge estimates only included indirect in-hospital costs. As a result, our charge estimates are likely underestimations of the true financial impact of these devastating infections. Third, this study included procedures that were performed prior to 2003. Since this time, greater emphasis has been placed on appropriate peri-operative antibiotic administration; thus, rates of SSI due to MRSA or MSSA may have changed since 2003. Finally, most of the surgical procedures we examined were cardiothoracic and orthopedic procedures. Thus, our results may be more reflective of the outcomes of SSI due to MRSA in these types of procedures. In fact, patients with orthopedic procedures were less likely to have adverse outcomes than patients that underwent other types of procedures. Thus, inclusion of a high number of orthopedic procedures may have biased our results towards the null and led to an underestimation of the impact of SSI due to MRSA on adverse clinical outcomes.

In summary, our study provides novel and interesting data regarding the clinical and financial impact of SSI due to MRSA and the impact of methicillin resistance among patients with SSI due to *S. aureus*. Not surprisingly, SSI due to MRSA led to incredibly poor outcomes compared to uninfected controls. Of particular interest, methicillin-resistance led to a longer duration of hospitalization and increased healthcare costs but did not increase the risk of mortality among patients with SSI due to *S. aureus*. Our estimates for the financial impact of SSI due to MRSA can be used to determine the cost-effectiveness of preventative strategies.
